# Effects of Visual Cues of Object Density on Perception and Anticipatory Control of Dexterous Manipulation

**DOI:** 10.1371/journal.pone.0076855

**Published:** 2013-10-16

**Authors:** Céline Crajé, Marco Santello, Andrew M. Gordon

**Affiliations:** 1 Department of Biobehavioral Sciences, Teachers College, Columbia University, New York, New York, United States of America; 2 School of Biological and Health Systems Engineering, Arizona State University, Tempe, Arizona, United States of America; VU University Amsterdam, The Netherlands

## Abstract

Anticipatory force planning during grasping is based on visual cues about the object’s physical properties and sensorimotor memories of previous actions with grasped objects. Vision can be used to estimate object mass based on the object size to identify and recall sensorimotor memories of previously manipulated objects. It is not known whether subjects can use density cues to identify the object’s center of mass (CM) and create compensatory moments in an anticipatory fashion during initial object lifts to prevent tilt. We asked subjects (n = 8) to estimate CM location of visually symmetric objects of uniform densities (plastic or brass, symmetric CM) and non-uniform densities (mixture of plastic and brass, asymmetric CM). We then asked whether subjects can use density cues to scale fingertip forces when lifting the visually symmetric objects of uniform and non-uniform densities. Subjects were able to accurately estimate an object’s center of mass based on visual density cues. When the mass distribution was uniform, subjects could scale their fingertip forces in an anticipatory fashion based on the estimation. However, despite their ability to explicitly estimate CM location when object density was non-uniform, subjects were unable to scale their fingertip forces to create a compensatory moment and prevent tilt on initial lifts. Hefting object parts in the hand before the experiment did not affect this ability. This suggests a dichotomy between the ability to accurately identify the object’s CM location for objects with non-uniform density cues and the ability to utilize this information to correctly scale their fingertip forces. These results are discussed in the context of possible neural mechanisms underlying sensorimotor integration linking visual cues and anticipatory control of grasping.

## Introduction

Skilled grasping and object lifting is dependent on both anticipatory planning and feedback mechanisms. Due to delays in sensory feedback, the initial fingertip force development following grasp contact relies on anticipatory planning processes. Anticipatory force planning is based on both visual cues about the object’s physical properties and sensorimotor memories of previous actions associated with grasped objects [1]–[6]. Specifically, vision can be used to estimate object mass based on the object size [3], [7], [8] and density [3], [9] to identify and recall sensorimotor memories of previously manipulated objects [3].

Subjects can use visual geometry cues to identify an object’s center of mass (CM) [10]–[14]. Subjects are able to use visual object geometry also to partition the forces between opposing digits to generate a compensatory moment to prevent object tilt during lifting an object with an asymmetric mass distribution [15]. In contrast to object size and shape cues (e.g., [7], [8], [16], [17]), considerably less is known about the use of visual cues related to object density. For symmetric objects, visual density cues are appropriately used to scale the fingertip forces during initial lifts with novel objects, e.g., higher forces are used for metal than wood or plastic [9]. However, some objects we manipulate, including tools (e.g., hammers) consist of more than one material, and thus might have an asymmetric mass distribution. In such instances, it is not known whether subjects can use density cues to identify the object’s CM and create compensatory moments in an anticipatory fashion during initial object lifts to prevent tilt.

The present study was designed to address the following three questions. First, we asked whether subjects can use density cues to scale fingertip forces when lifting visually symmetric objects of uniform densities (i.e., symmetric CM). We hypothesized that subjects would be able to scale their fingertip forces when the density was uniform as previously described [9]. Second, we asked whether subjects are able to estimate CM location of objects with a symmetric shape and an asymmetric CM using uniform and non-uniform density cues. Since subjects can use object shape to identify object CM, we hypothesized that subjects can identify the approximate CM location based on density cues regardless of whether they are symmetrically distributed or not. Third, we asked whether subjects can use density cues to scale fingertip forces when lifting visually symmetric objects of non-uniform densities (i.e, asymmetric CM). We hypothesized that subjects would fail to generate compensatory moments to prevent tilt on initial lifts due to the incongruence between shape and density cues.

## Methods

### Subjects

Sixteen healthy subjects (3 males and 13 females, aged 20–34 years) participated in the study. All subjects were right handed, and had normal or corrected to normal vision. Subjects provided informed written consent prior to the experiment, and were naive to the purpose of the study. Written informed consent was obtained from subjects prior to testing in accordance with the Declaration of Helsinki and the study was approved by the Teachers College, Columbia University Institutional Review Board.

### Apparatus

**Figure 1:**
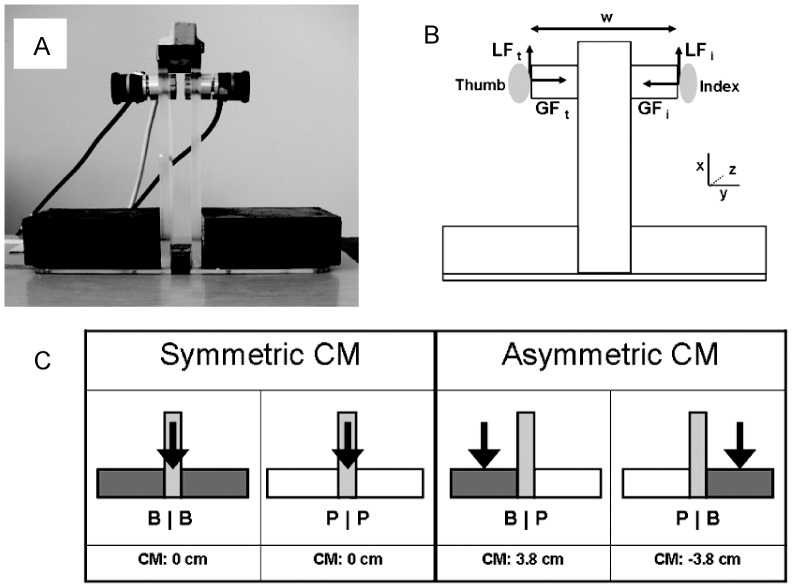
Experimental set-up. A. The inverted T-shaped apparatus. Here the mass distribution is hidden by the balsawood covers. B. Schematic drawing of the device, with w representing the distance between the fingers (w = 8 cm). Load forces (LF, parallel to the grip surface) and grip forces (GF, perpendicular to the grip surface) were measured. T = thumb, I = index. C. Configurations of the bars on the grip device used in the experiments 1 and 2. The arrows represent the location of the center of mass (CM). B = brass bar, P = plastic bar.

The apparatus used consisted of two parallel grip surfaces (diameter = 17 mm, distance between grip surfaces = 8 cm), mounted on an inverted T-shaped object made of Plexiglas (115 grams; Figure 1). The grip surfaces covered two force sensors (Nano 17 F/T transducer, ATI Industrial Automation, NC) that measured grip force and load force applied by each finger (with a resolution of 0.05 N and 0.025 N, respectively). We used an electromagnetic position-angle sensor (Polhemus Fasttrack, 0.05° resolution) mounted on the top of the object to measure its vertical position and tilt. The weight of the entire apparatus, including the force sensors and position-angle sensor, was 165 g.

Two 7.5 cm^3^ bars were placed along the horizontal base of the object. The bars consisted of two different materials: hollow white plastic (P; mass = 15 g) and solid brass (B; mass = 405 g). As we were interested in how subjects use visual cues about object density to scale grip forces in an anticipatory fashion, we manipulated the configuration of the plastic/brass bars placed at the bottom of the object. This resulted in 4 possible object configurations: B|B (symmetric mass distribution, heavy), P|P (symmetric mass distribution, light), B|P (asymmetric mass distribution, center of mass, CM, on thumb side) and P|B (asymmetric mass distribution, CM on index finger side) (Figure 1C). For object configurations with an asymmetric CM, the asymmetric mass distribution (CM located 3.8 cm from the object’s center) resulted in a clockwise and counterclockwise moment in the *xy* plane of −15.1 Ncm and 15.1 Ncm for P|B and B|P, respectively). Note that the object shape was always the same (symmetric inverted T), regardless of the combination of B and P bars. Therefore, subjects had to use visual cues about the bars’ density to estimate the object mass and the CM location to scale fingertip forces to the object mass and mass distribution in an anticipatory fashion.

### Procedures

Eight subjects performed two tasks:

#### Lifting task

Subjects were seated in front of a table with the experimental setup. The apparatus was placed 40 cm in front of the subjects’ right shoulder. Before each trial, subjects placed their right hand with the palm down at the start location at the table. Subjects were asked to grasp the object with the thumb and index finger and lift the object ∼15 cm, hold it there for several seconds and then replace and release the object back on the table. They were instructed to keep the bottom of the object parallel to the table (i.e., to prevent object tilt), and perform the movement at a self-selected speed. Verbal cues were provided as start and end signals for timing purposes. Five consecutive lifts of each object configuration (see below) were performed to measure effects of practice. For each object configuration, lifts were performed with density cues (object materials and their distributions were visible), and without density cues (object materials and distribution occluded). In the latter ‘no vision’ conditions a black, light-weight (<5 g) balsawood surface covered the brass and plastic bars, and thus no visual information was available about the object mass distribution. Therefore, the ‘no vision’ conditions had the same geometry cues (symmetric inverted T-shape) as the vision conditions, but lacked density cues. As such, in the ‘no vision’ conditions the object looked symmetrical both in configuration and density. This experimental condition served as a control whereby performance on initial lifts with density cues visible could be compared to initial lifts when they were not visible.

#### Center of mass estimation task

Before and after lifting each objects with asymmetric mass distributions (above) 5 times, subjects were asked to indicate the location of the object CM in the horizontal plane to assess their ability to estimate object mass distribution. For this task, the object was placed behind a ruler, and subjects pointed with a pencil at the location where they estimated object CM, which was recorded by the experimenter. Note that when this task was performed *before* lifting the object, subjects had to use visual cues about object geometry and density. In contrast, when subjects had to estimate object mass distribution *after* having lifted the object 5 times, they could integrate visual cues with sensorimotor memories [3], [7], [8]. This task was also performed during blocks of trials with no vision (balsawood covers, described above) to determine whether experience lifting the object would lead to modify subjects’ initial estimation of CM location.

Thus, subjects always first estimated the object’s CM location, then lifted the object 5 times, and then estimated the object’s CM location again. The time between the 5 successive lifts was ∼5 s, and the time between each object configuration (i.e., between the 5th lift of one condition and the first lift of another, including the pointing task) was ∼1 min.

#### Hefting task

Hefting whole objects prior to lifting has been shown to “calibrate” the sensorimotor system [18]. Thus, hefting individual object parts before lifting the object might result in improved performance on initial lifts of objects with an asymmetric mass distribution. To test this possibility, 8 additional subjects who did not participate in the above tasks first held their hand with their palm facing up while the inverted T frame, the brass and plastic bars were individually placed in the subject’s hand in a random order. Subjects held them for approximately 10 s before they were removed from their hand. As such, they had information about the mass of the individual parts before starting the experiment. Following this procedure, these subjects performed 5 lifts of the asymmetric P|B and B|P conditions as described above.

### Data Processing

During the experiment, fingertip forces applied to the force transducers and position data of the apparatus were sampled at 400 and 120 Hz respectively, using SC/Zoom (Umeå University, Sweden) and analyzed using custom written software in Matlab. The data were filtered using a second order dual low pass Butterworth filter with a 6 Hz cutoff. The following temporal and spatial variables were computed:

1. *Time of lift onset* was defined as the first time point at which the vertical velocity of the object exceeded above 5 mm/s and subsequently remained above this value for at least 250 ms.
2. *Object tilt* was defined as the angle between the gravitational vertical and the vertical axis of the object in the *xy* plane. We focused on the initial peak tilt that occurred shortly (∼125 ms) after lift onset, i.e., before corrective responses to counter object tilt can be made [19], [20]. Positive and negative values represent clockwise (towards the index finger) and counterclockwise (towards the thumb) tilts, respectively.
3. *Load force* (LF) is the vertical tangential force component produced by the digits to lift the object. Load forces of each digit at the time of lift onset were used to compute the net moment (*compensatory moment*) generated by the subjects at object lift onset (see below).
4. *Maximum load force rate* was defined as the maximum of *d*LF/*d*t using the summed load forces of the two digits. The maximum load force rate before lift-off is informative for scaling of load forces to the object mass before proprioceptive information about the object mass is available at object lift [3], [5], [19], [20].
5. *Grip force* (GF) is the normal force component produced by the digits. Our analyses focused on the grip forces of both digits at the time of lift onset.
6. *Maximum grip force rate* was defined as the maximum of *d*GF/*d*t using the average grip forces of the two digits. The maximum grip force rate before lift-off is informative for scaling of grip forces to the object mass before proprioceptive information about the object mass is available.
7. *The compensatory moment* was defined as follows:

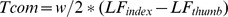



Where *w* is the grip width (8 cm), *LF_thumb_* and *LF_index_* are the load force generated by the thumb and index finger, respectively.

### Design and Statistical Analyses

Each research question had its own design and accompanying statistical analyses, as described below.

**Can subjects use density cues to scale fingertip forces when lifting symmetric objects (uniform densities)?** As proprioceptive information about the object mass is not available until lift-off, appropriate force scaling is required to scale the rate of load and grip force development before lift-off. To verify whether subjects were able to use density cues to scale fingertip forces as previously reported [9], we measured grip and load force scaling (force rates) when subjects lifted a heavy symmetric object (i.e, B|B) and a light symmetric object (P|P). Note that the specific configuration of the bars, and thus density cues, informed subjects about the different object masses. Both object configurations were lifted 5 times each. The order of lifting the heavy or the light object was alternated between subjects.

If subjects use visual information to scale digit forces to mass, we expected higher maximum force rates for the heavy symmetric objects than the light symmetric objects. Also, we were interested if experience with lifting the object affected force scaling before lift-off (thus comparing lift 1 and lift 5). Therefore we performed repeated measures ANOVA with *Object Mass* (2 levels; heavy versus light: B|B versus P|P), and *Practice* (2 levels; lift 1 and lift 5) as the within-subject factors for maximum grip force rate and maximum load force rate.

**Can subjects estimate object CM location of objects with an asymmetric CM using visual density cues (non-uniform densities)?** We were interested if subjects were able to estimate CM locations in objects with symmetric object geometry but an asymmetric mass distribution (i.e., B|P and P|B). Thus, in these object configurations, density cues provided subjects information about mass distribution. We tested if the estimation of object CM location was dependent on whether subjects 1) had visual information about the object configuration and 2) had experienced the object dynamics. Therefore subjects estimated the CM before and after 5 lifts of the objects with an asymmetric CM. For comparison, we also included ‘no vision’ conditions where the brass and plastic bars were covered with the balsawood covers. While we expected subjects to estimate the CM location to be centered prior to lifts of the objects with the asymmetric CM, we were mainly interested in how that perception changed following the 5 lifts, and whether this experience alone allowed accurate CM location estimation. To investigate effects of having visual information about object mass distribution and effects of practice, we performed repeated measures ANOVAs with *Practice* (2 levels; lift 1 and lift 5) and *Vision* (2 levels; vision versus no vision) as the within-subject factors on the estimated CM locations, separately for B|P and P|B.

**Can subjects use non-uniform object density cues to modulate compensatory moment to object CM (asymmetric mass distribution)?** To test if subjects could use object density cues to modulate the compensatory moment to object CM, subjects lifted objects with symmetric object geometries (inverted T) and asymmetric mass distributions (i.e., B|P and P|B) for 5 consecutive times. We also included conditions where no visual information was available about the object mass distribution (balsawood covers) for comparison to determine whether subjects benefited from visual information about mass distribution on the first lift, and whether vision facilitated learning over subsequent lifts compared to lifting experience only (no vision). Thus in summary, there were 4 conditions (B|P vision, B|P no vision, P|B vision, and P|B no vision), each with 5 lifts, resulting in 20 trials. The order of conditions was counterbalanced across subjects.

To analyze the effects of availability of visual cues about object density and experience with lifting the object, repeated measures ANOVAs with *Practice* (2 levels, lift 1 and lift 5) and *Vision* (2 levels, vision versus no vision) as the within-subject factors were performed for the variables Object Tilt and Compensatory Moment, separately for the asymmetric configurations B|P and P|B. For all research questions, comparisons of interest were further analyzed using planned comparisons with Bonferroni corrections.

## Results

### Can Subjects use Density Cues to Scale Fingertip Forces when Lifting Symmetric Objects (Uniform Densities)?

First, we were interested if subjects were able to scale the grip and load forces *when lifting symmetric* objects of different densities (and thus masses), as was previously found by [9]. Therefore, subjects lifted the object configuration B|B and P|P five times consecutively. If subjects use visual information about object density to scale digit forces to mass, we expected higher maximum grip and load force rates for the heavy (B|B) objects.

**Figure 2:**
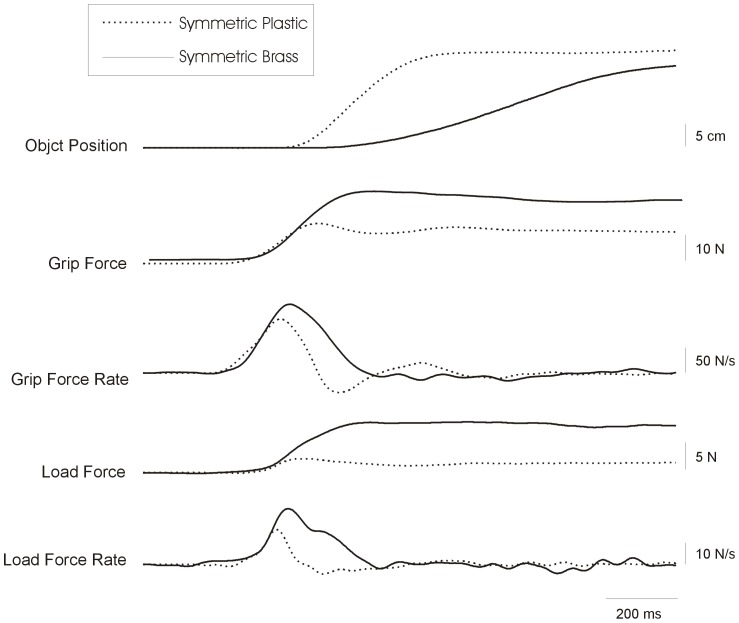
Object vertical position (cm), grip forces (N), grip force rate (N/s), load force (N) and load force rate (N/s) from a representative subject lifting an object in the symmetric condition P|P and B|B for the first time, respectively.

**Figure 3:**
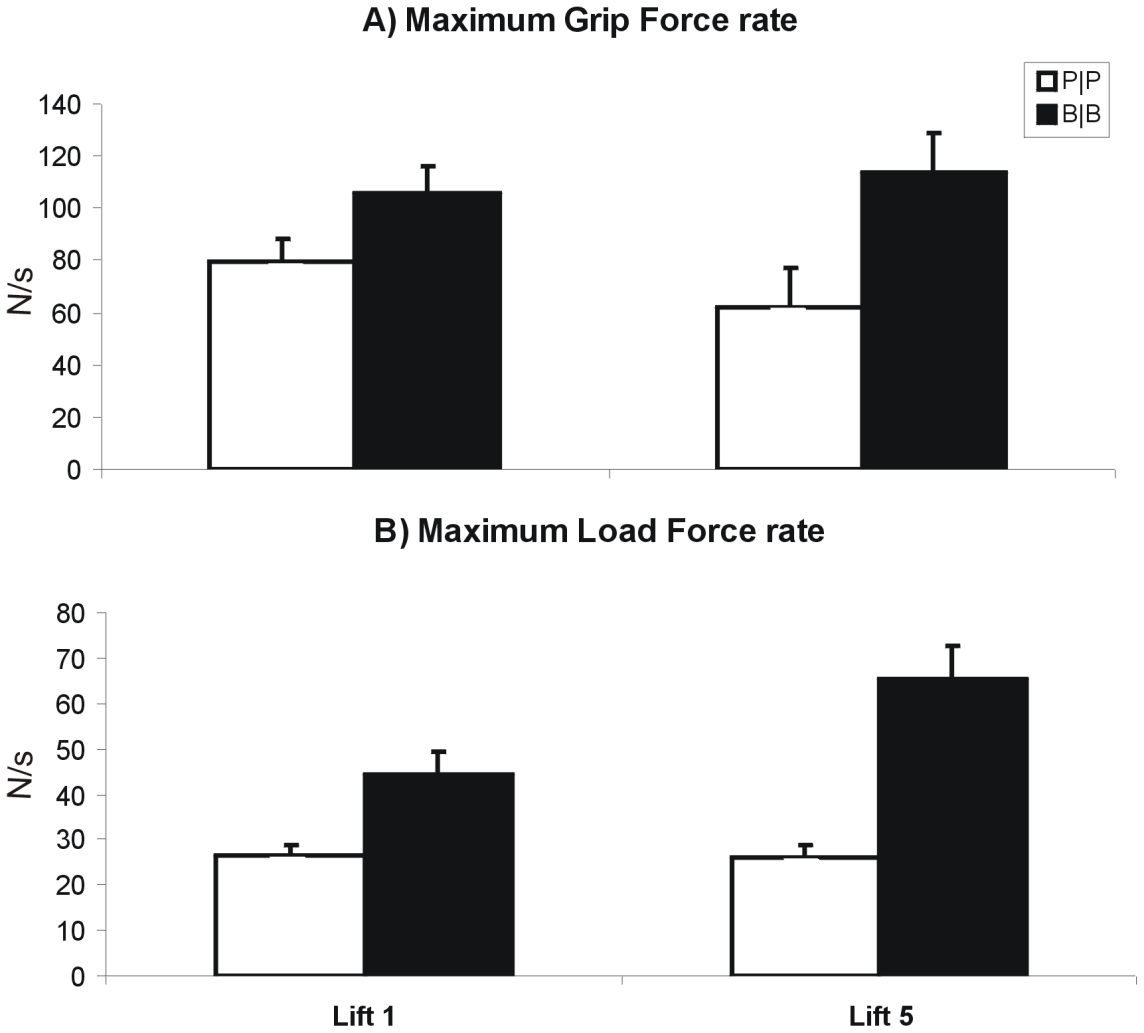
Mean and SEM of the maximum grip force rate (A) and maximum load force rate (B) in lift 1 and 5 for the heavy object (B|B, grey bars) and the light object (P|P, white bars).

Figure 2 shows force and position traces from a representative subject lifting each object with a symmetric mass distribution (B|B and P|P) for the first time. Overall, the rates of grip and load force increase were higher for the heavier (B|B) object than the lighter (P|P) object. Across all subjects, subjects applied higher grip and load force rates prior to lifting the heavy object compared with the light object already on the first lift (main effect of *Object Mass*, *F*(1,7) = 39.58, p<0.01, *η^2^* = 0.85 and *F*(1,7) = 21.50, p<0.01, *η^2^* = 0.75 for maximum load and grip force rate, respectively; see Fig. 3A and B). The maximum load force rate increased from lift 1 to lift 5 (main effect of *Practice*, *F*(1,7) = 5.67; *p*<0.05, *η^2^* = 0.45), however this effect was only significant for the heavy object (interaction effect of *Object Mass*×*Practice, F*(1,7) = 9.65, p<0.05, *η^2^* = 0.58, post hoc planned comparison, *p*<0.01). There were no effects of practice on the grip force rate for either mass. These findings suggest that subjects could use density cues for an object with a symmetrically distributed mass to scale the fingertip force increase on the first lift, with subtle fine-tuning following practice.

### Can Subjects Estimate Object CM Location of Objects with an Asymmetric CM Using Visual Density Cues (Non-uniform Densities)?

**Figure 4:**
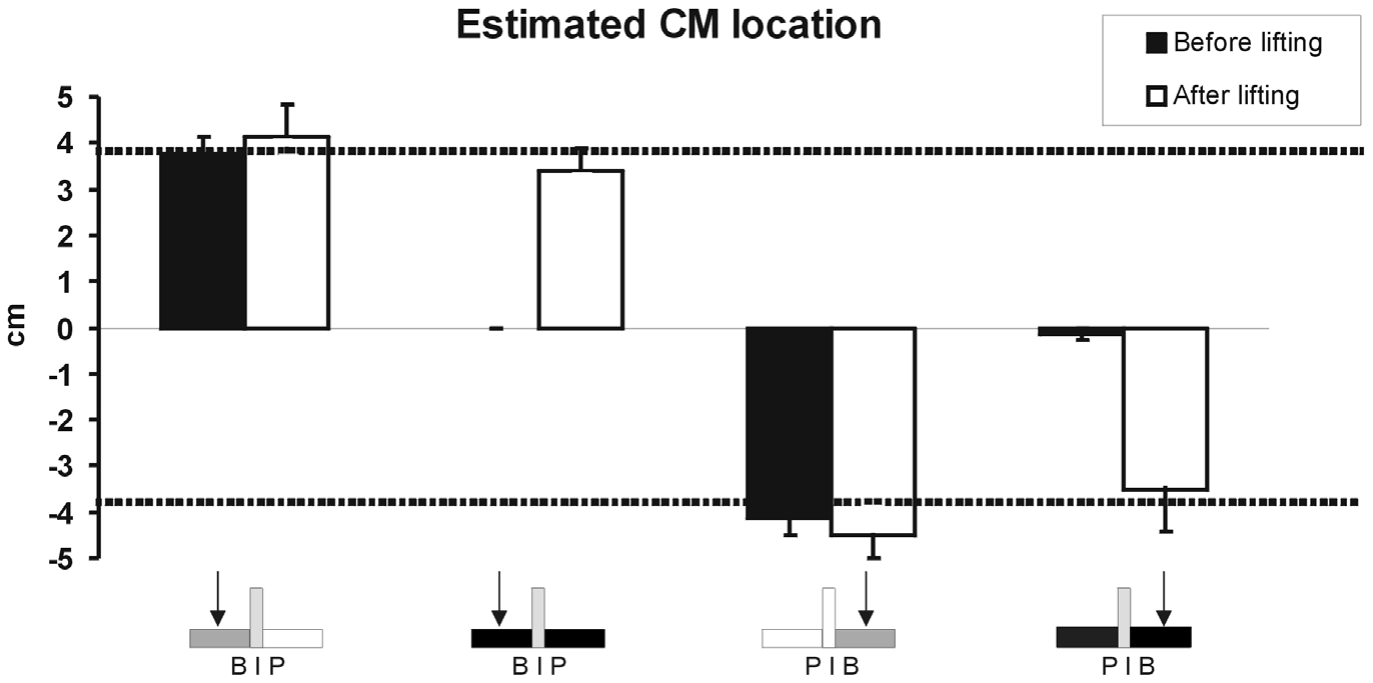
Mean and SEM estimated CM location for uncovered B|P (first column), covered B|P (second column), uncovered P|B (third column), and covered P|B (fourth column) for the first and fifth lift (black and white bars, respectively). Zero represents the objects’ midline, negative values represent a CM estimate on the index finger side, and positive values represent a CM estimate on the thumb finger side. The dotted horizontal lines represent the actual CM locations in the asymmetric configurations.

In the vision conditions, prior to lifting objects with an asymmetric CM, subjects were very accurate in estimating the CM location prior to the initial lift when they had visual information about the object configuration (see Figure 4, black bars). As seen in the figure, on average, they were within <1 mm of the actual CM location. Since they were already so accurate prior to lifting the object in the vision condition, there were no statistically significant differences in estimated CM before and after lifting the object (effect of practice, p>0.05). In the no vision (covered) conditions, as expected, subjects initially estimated the CM to be centered (symmetrical mass distribution) before lifting (see Figure 3), resulting in an inaccurate CM estimation (main effects of *Vision*, *F*(1,7) = 24.65, *p*<0.0, *η^2^* = 0.781 for P|B and *F*(1,7) = 53.84, *p*<0.01, *η^2^* = 0.89 for B|P). Importantly, after having lifted the object 5 times (and thus having experience with object dynamics) the estimated location of CM was closely aligned (within ∼1 mm) with the actual CM location, and similar to the CM estimations in the vision condition (main effects of *Practice*, *(F*(1,7) = 11.01, *p*<0.05, *η^2^* = 0.61 for P|B and *F*(1,7) = 12.81, *p*<0.01, *η^2^* = 0.65 for B|P) (Figure 4). Thus, subjects accurately estimated the object’s CM when visual cues related to density are available. When density cues are unavailable, subjects initially relied purely on the object’s geometry as expected, but their estimation becomes quite accurate after experience with lifting the object.

### Can Subjects use Non-uniform Object Density Cues to Modulate Compensatory Moment to Object CM (Asymmetric Mass Distribution)?

**Figure 5:**
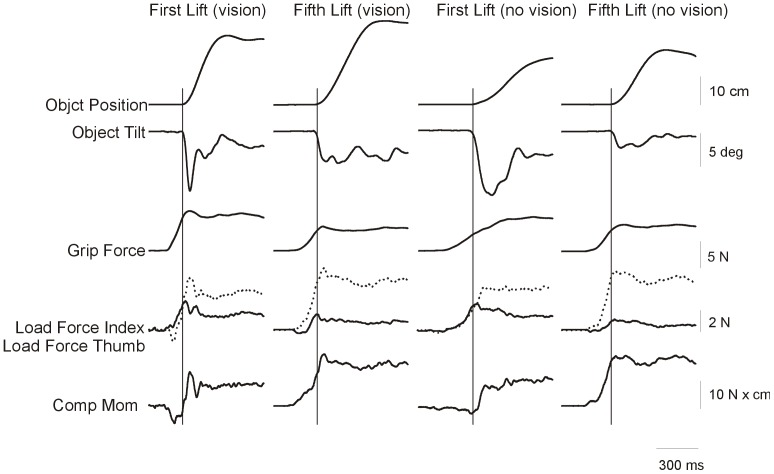
Object vertical position (cm), Object tilt (degrees), Grip forces (N, solid line for the thumb, and dotted line for the index finger), Load forces (N, solid line for the thumb, and dotted line for the index finger and Compensatory moment (N×cm) from a representative subject lifting an object in the condition P|B, thus plastic on the left (thumb) side and brass on the right (index finger) side. The columns represent data from the first and fifth lift of the vision condition, and the first and fifth lift of the no vision condition, respectively. The vertical grey lines represent the time of lift onset.

Force and position traces from a representative subject lifting the object with an asymmetric mass distribution are presented for four lifts for the P|B configuration in Figure 5. The first two columns represent the data for the first and fifth lift in the vision condition (thus subjects had visual information about the mass distribution). The third and fourth columns represent the data of the first and fifth lift in the no vision condition, where the covers were used (i.e., subject had no visual information about the mass distribution). The grip and load forces increased before lift onset until the load forces exceeded the gravitational forces and the object it lifted from the table (vertical lines). In the first lift of both the ‘vision’ and ‘no vision’ condition, there was little compensatory moment generated at the point of object lift-off. Consequently, a clockwise tilt of the object initially occurred as the object was lifted. Thus, the subject was unable to anticipate the object’s CM to prevent object tilt, even when information about the object’s mass distribution was available (i.e., in the vision conditions). However, after an initial tilt, the subject attempted to correct the tilt by adapting the load forces (increase the load force of the index finger and decrease the load force of the thumb) creating a compensatory moment. In contrast, when lifting the object for the fifth time, the subject anticipated the objects mass distribution by scaling the load forces of the thumb and index finger (and thus creating a compensatory moment) before lift onset, resulting in a minimal object tilt after lift onset. Overall as seen below these findings are representative of all subjects we tested.

**Figure 6:**
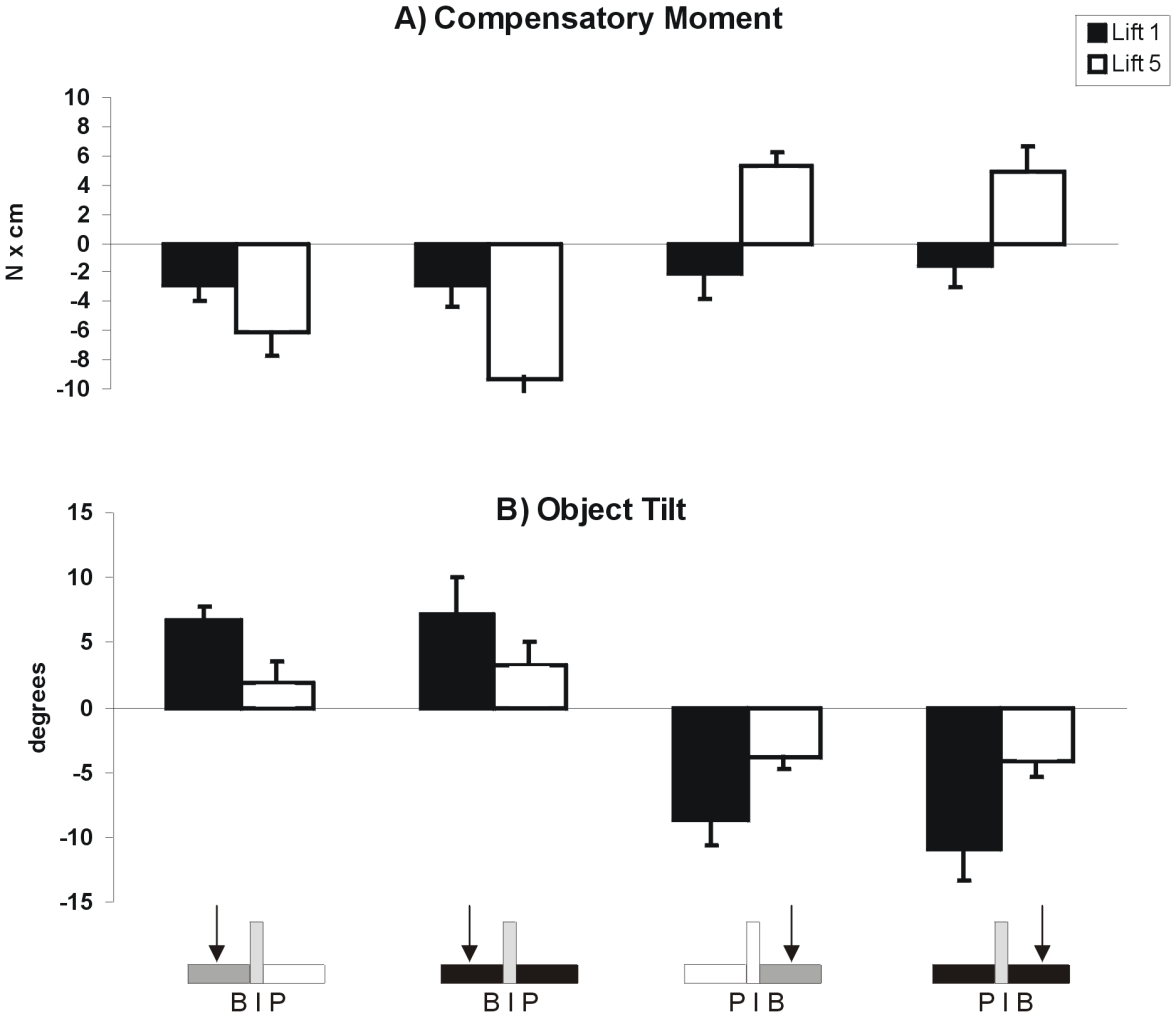
Compensatory moment (A) and Object Tilt (B) for uncovered B|P (first column), covered B|P (second column), uncovered P|B (third column), and covered P|B (fourth column) for the first and fifth lift (black and white bars, respectively). For compensatory moment: negative values indicate moment in a clockwise direction (i.e., larger LF applied by the thumb than index finger), whereas positive values indicate compensatory moment in a counter clockwise direction (i.e., larger LF applied by the index finger than thumb). Positive and negative values represent clockwise (towards the index finger) and counterclockwise (towards the thumb) tilts, respectively.

Despite being able to correctly indicate the direction of the CM location before lifting the object (research question 2, above), when lifting the asymmetric configurations (B|P and P|B) for the first time, both in the ‘vision’ and ‘no vision’ condition, subjects did not exert an appropriate compensatory moment, and therefore could not prevent object tilt. However, after 5 lifts, they learned to create a compensatory moment, thus leading to a significantly smaller object tilt (see Figures 6 A and B) (main effect of *Practice* for Object Tilt (*F*(1,7) = 9.41, *p*<0.05, *η^2^* = 0.57 for B|P and *F*(1,7) = 27.09, *p*<0.01, *η^2^* = 0.80 for P|B), and main effect of *Practice* for Compensatory Moment (*F*(1,7) = 10.17, *p*<0.05, *η^2^* = 0.59 for B|P and *F*(1,7) = 18.16, p<0.05, *η^2^* = 0.72 for P|B). There were no significant interaction effects between *Visio*n and *Practice* (Object Tilt: F(1,7) = 0.046, *p = *0.84, *η^2^* = 0.007 for B|P and *F*(1,7) = 0.65, p = 0.45, *η^2^* = 0.08 for P|B, Compensatory Moment: *F*(1,7) = 0.82, *p = *0.40, *η^2^* = 0.11 for B|P and *F*(1,7) = 0.034, *p* = 0.86 for P|B, *η^2^* = 0.005).

**Figure 7:**
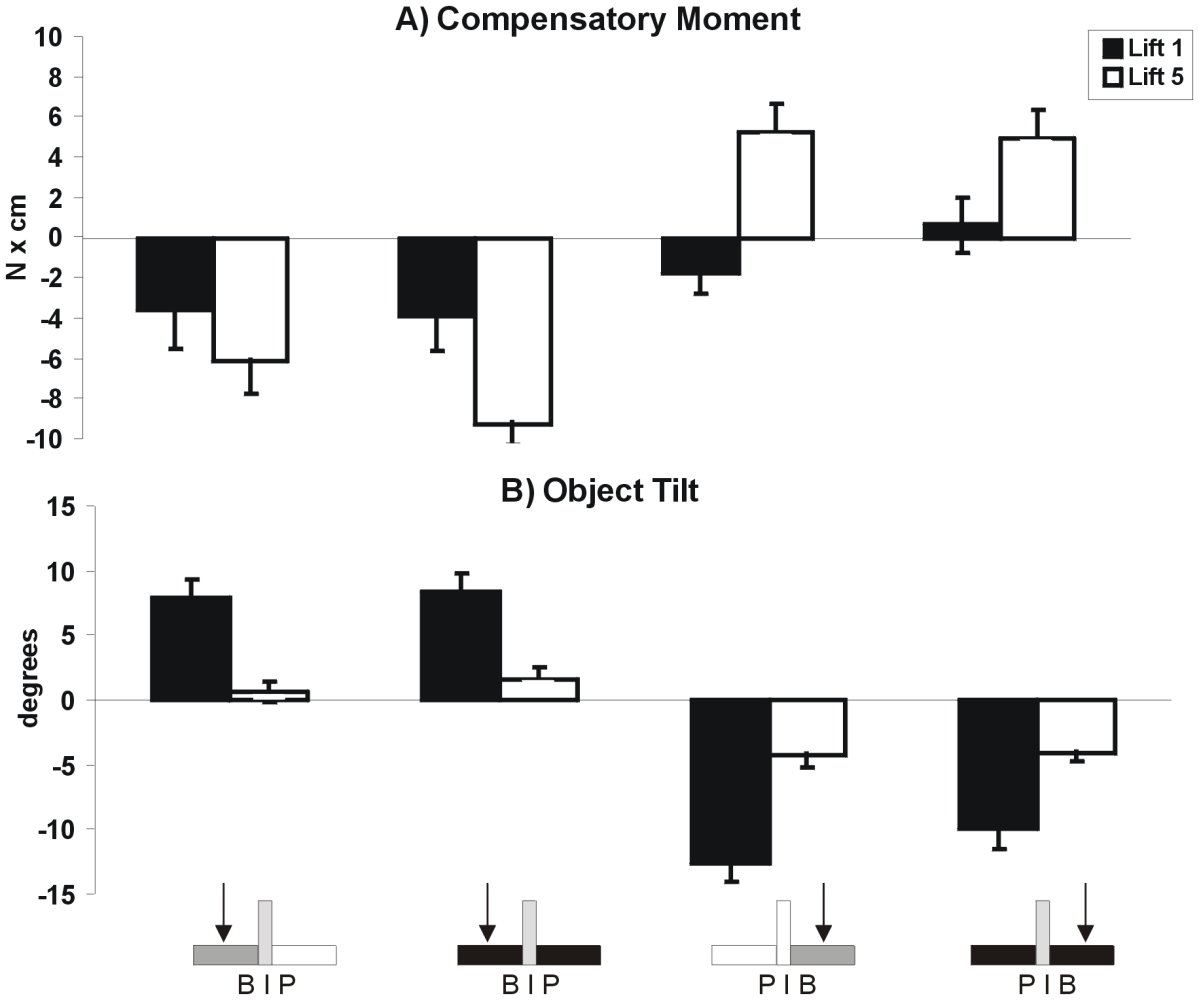
Compensatory moment (A) and Object Tilt (B) for uncovered B|P (first column), covered B|P (second column), uncovered P|B (third column), and covered P|B (fourth column) for the first and fifth lift (black and white bars, respectively after subjects had lifted the individual object parts.

As described above, subjects could use density cues for scaling fingertip forces when lifting symmetric objects (uniform density cues) and to estimate the CM location of objects with an asymmetric CM using visual density cues (non-uniform density cues), but not for scaling grip forces to modulate a compensatory moment to object CM (asymmetric mass distribution) (research question 3). Hefting whole objects prior to lifting has been shown to “calibrate” the system [18]. To test whether hefting individual object parts before lifting the object might result in improved performance in the third task (i.e., lifting objects with an asymmetric mass distribution), we investigated research question 3 in a new group of subjects (n = 8), and had them heft the different parts of the object (i.e., brass cube, plastic cube and the apparatus) once with the same (right) hand before performing the experimental lifting trials. As seen below, having this recent experience with the objects parts did not result in differential effects as reported for research question 3 (compare figure 6 and 7).

Similar to the findings of research question 3, subjects could not use density cues to create a compensatory moment to prevent object tilt in the first lift. However, in the fifth lift they had learned to anticipate the asymmetric mass distribution by creating a compensatory moment (Object Tilt: main effect of *Practice*, *F*(1,7) = 26.93, *p*<0.01, *η^2^* = 0.79 for B|P and *F*(1,7) = 57.71, *p*<0.01, *η^2^* = 0.89 for P|B; Compensatory Moment: main effect of *Practice*, *F*(1,7) = 10.83, *p*<0.05, *η^2^* = 0.61 for B|P and *F*(1,7) = 36.37, *p*<0.01, *η^2^* = 0.84 for P|B). There were no significant *Vision*×*Practice* interactions (Object Tilt: *F*(1,7) = 1.14, *p = *0.32, *η^2^* = 0.14 for B|P and *F*(1,7) = 0.036, *p* = 0.86 for P|B, *η^2^* = 0.005, Compensatory Moment: *F*(1,7) = 0.77, *p = *0.41, *η^2^* = 0.10 for B|P and *F*(1,7) = 3.54, *p* = 0.10, *η^2^* = 0.34 for P|B).

To determine if recent experience with hefting the different object parts aided performance in any way, we compared compensatory moment at lift-off and subsequent peak tilt in the first and fifth lift in the experiments with and without hefting prior to the experimental lifts using a 2 (hefting vs. no hefting)×2 (vision vs. no vision)×2 (1^st^ vs. 5^th^ lift) ANOVA, with repeated measures on the last two factors. Hefting did not improve performance for Compensatory Moment (*F*(1,14) = 2.45, *p* = 0.14, *η^2^* = 0.15 for B|P and *F*(1,14) = 2.95, *p* = 0.11, *η^2^* = 0.17 for P|B) or Object Tilt (*F*(1,14) = 0.002, *p* = 0.97, *η^2^* = 0.000 for B|P and *F*(1,14) = 0.012, *p* = 0.91, *η^2^* = 0.001 for P|B). Furthermore, similar to the hefting and non-hefting results reported above, in the combined data set (n = 16), when lifting the asymmetric configurations (B|P and P|B) for the first time, both in the ‘vision’ and ‘no vision’ condition, subjects did not exert an appropriate compensatory moment, and therefore could not prevent object tilt. However, after 5 lifts, they learned to create a compensatory moment, thus leading to a significantly smaller object tilt (main effect of *Practice* for Object Tilt (*F* (1,14) = 48.31, *p*<0.01, *η^2^* = 0.78, for B|P and *F*(1,14) = 45.27, *p*<0.01, *η^2^* = 0.76 for P|B), and main effect of *Practice* for Compensatory Moment (*F*(1,14*) = *20.73, *p*<0.01, *η^2^* = 0.60 for B|P and *F*(1,14) = 50.08, p<0.01, *η^2^* = 0.78 for P|B). There were no significant interactions between *Visio*n and *Practice* (Object Tilt: *F*(1,14) = 0.50, *p = *0.49, *η^2^* = 0.034 for B|P and *F*(1,14) = 0.074, *p* = 0.79, *η^2^* = 0.005 for P|B, Compensatory Moment: *F*(1,14) = 0.07, *p = *0.80, *η^2^* = 0.005 for B|P and *F*(1,14) = 0.86, *p* = 0.37, *η^2^* = 0.058 for P|B) or between groups (hefting and non-hefting) and any factor. Thus hefting did not improve performance, and the added statistical power associated with doubling the sample size did not alter the findings in any way.

## Discussion

The present study indicates that, consistent with other studies, when the mass distribution is uniform and object size is not varied, subjects used visual density cues to scale their fingertip forces in an anticipatory fashion. Second, our study indicates that subjects are able to accurately estimate an object’s center of mass based on visual density cues for objects with non-uniform mass distribution prior to ever having lifted it. However, despite their ability to estimate CM location prior to lifting when object density is non-uniform, subjects were unable to scale their fingertip forces to create a compensatory moment and prevent tilt on initial lifts. This suggests a dichotomy between the ability to accurately identify the object’s CM location for objects with non-uniform density cues and the ability to utilize this information to correctly scale their fingertip forces. These results are discussed in light of object features which allow or do not allow anticipatory grasp control (geometry and asymmetrical density, respectively) and potential neural mechanisms underlying sensorimotor integration linking visual cues and anticipatory grasp control.

### Dichotomy between Identification of Object CM and Anticipatory Digit Force Modulation

Subjects’ ability to accurately estimate CM location based on visual estimation of object density distribution prior to lifting them is consistent with studies showing they can use object geometry to identify object CM [10]–[14]. Experience with lifting the objects did not improve their already accurate estimation ability (see Figure 4). However, the presence of visual density cues was of no greater assistance to our subjects in generating a compensatory moment on the initial trials than when the cues were obscured (covered trials) and non-uniformly distributed (plastic/brass). Similarly, hefting object parts beforehand did not affect this ability. The dichotomy between accurate identification/estimation of object CM and action performance (failure to generate a compensatory moment to prevent tilt) also occurs when an object with an asymmetric CM distribution but symmetric appearance is rotated or translated after several lifts [19]–[23]. Thus, subjects’ awareness of the new CM location does not result in the ability to generate a compensatory moment that is appropriate to prevent tilt.

The results pointing to a dissociation between estimation of object features and motor actions are consistent with findings from patient (DF) with visual form agnosia, who is unable to perceive simple shapes, but is able to scale the aperture of the hand based on object size during reach-to-grasp [24]. They are also consistent with visuo-motor illusions such as the size-weight (e.g., [7], [8], [16], [25], [26]), the Ebbinghaus illusion [27], and material-weight illusion [9], [28], [29]. For example, after repeated lifts, subjects correct their initial erroneous sensorimotor predictions although they continue to experience the perceptual illusion [26], [30]. These studies, however, point to *inaccurate perception*, but *correct action* (cf. [31], [32] for studies where the dissociation does not exist). Thus, perceptual and sensorimotor representations may be functionally independent from one another [16], [33]. As estimation of object CM was accurate in our task, our interpretation is that the present motor errors could have only occurred at the sensorimotor transformation or execution level.

When object geometry is inconsistent with mass distribution (i.e., symmetric geometry, asymmetric mass distribution), visual geometry cues may override explicit knowledge of mass distribution. Specifically, our experience with objects consisting of only one material may make the CNS more dependent on shape than density cues. Nevertheless, as evidenced from lifts of different uniform-density objects in the present and other studies [9], density cues certainly can influence planning and execution of grasping. However, in a study of the size-weight illusion across objects of various densities [33], it was shown that a fixed increase in size yielded a fixed increase in expected weight, regardless of apparent density. Thus, visual cues about object geometry may be utilized to a greater extent than those about object density. However, in our study using an object invariant in size but varying in density and mass distribution, subjects learned to create a compensatory moment equally well over repeated lifts regardless of whether visual cues about density distribution were present, or if the object appeared to have a symmetric density but in fact did not (covered lifts with an asymmetric CM location). Thus, object geometry cues do not override the learned behavior based on sensorimotor memories of the actual mass distribution after repeated lifting. It should be noted that consistent with other learning studies of lifting objects with an asymmetric mass [19]–[23], even after 5 lifts, the compensatory moment did not perfectly counter the asymmetric mass distribution, and a tilt occurred. However, there was approximately a 3-fold improvement across conditions from the first to fifth lift (Figs. 6 and 7), and subjects may have deemed the small tilt as “good enough” since there was no consequent of imprecision.

The primary motor cortex (M1) appears to be involved in adaptation of grasping forces to object weight [34] and integrating sensorimotor memories with current visual information. For example, using repetitive magnetic transcranial stimulation (rTMS) [6], found that M1 stores a sensorimotor memory of object weight by changing the level of excitability of the involved muscle representations. This representation was suppressed ∼150 ms after object presentation when visual information is available. Thus, M1 may be involved in linking action planning with memory of object properties, and this linkage is sensitive to visual cues of object size. Our results suggest that such linkage allows for effective use of visual cues to plan action correctly (i.e., in this case, symmetry). Sensory areas in the parietal and temporal cortices may also be responsive to the size and/or shape of objects to be manipulated. For example, the left anterior intraparietal area, left superior-parietal lobule, and fusiform gyrus have been shown to be responsive to object size [34]. It has been suggested that these areas may be involved in the sensorimotor transformation of visual information about object geometry into motor commands specifying the movements needed to manipulate the objects [34]. Finally left ventral premotor area (PMv) has been shown to be involved in judging the weight of objects when subjects observe a video of an object being lifted [35], [36] as well as being responsive to the density of objects to be lifted [34]. Given its connections with numerous brain areas [37], PMv may integrate various sensory information for higher-level processing. In summary, a highly distributed network of cortical areas is involved in integrating sensorimotor memories with visual perception of object features for action planning. Our results suggest a competition within such network whereby subjects either cannot initially use density cues indicating asymmetric mass distribution despite their accurate perception, or geometry cues (symmetry) override them until they experience object lifts, creating accurate sensorimotor memories dominating subsequent lifts.

### Factors Allowing or Interfering with Vision-based Anticipatory Grasp Control

The inability to use visual density cues for objects with an asymmetric CM is at odds with the findings with other studies showing that vision can be used to scale fingertip forces based on specific object properties. For example, subjects can scale fingertip forces in anticipation of the weight of an object based on its size [7] even during the first encounter with that object. Similarly, subjects appear capable of associating the density with the required fingertip force output when the density is uniform [30], [33], (see present results). Furthermore, vision cues regarding object shape are used to determine the required fingertip force scaling [38].

Interestingly, similar findings have been reported in object transfer studies, whereby after several lifts, subjects are required to re-lift the same object in a new location or configuration (e.g., rotated) or with the contralateral hand. For example, following several lifts with an object, subjects demonstrate correct fingertip force scaling when lifting an object of symmetric weight or texture with the contralateral hand [39], [40]. In contrast, for objects requiring asymmetric distribution of fingertip forces due to different textures at the thumb and index finger [41] or asymmetric mass distribution [10], [20], [22], [23], [42], subjects fail to transfer learned force scaling if the object is rotated 180° or translated to the contralateral hand without lifting it despite subjects’ awareness of the new CM location. Such failure to generalize learned behaviors is not confined to studies of fingertip forces during grasping. For example, it can be seen during a bimanual object-manipulation task in which subjects grasped two handles attached by a virtual elastic band, moving the right hand to stretch the band while attempting to hold the contralateral hand and still generating compensatory forces [43]. Specifically, when the visible band directly connected the two handles, subjects produced compensatory forces in the appropriate direction. However, when the elastic band connected the handle via a pulley (altering the required direction of the compensatory force), subjects failed to generate compensatory forces in the correct direction.

Why can vision be used in some cases to signal the required forces/actions and not others? The findings across the above studies may suggest that symmetric and asymmetric objects (or actions) challenge the system differently. Specifically, subjects appear to be able to scale the overall gain of fingertip forces to object properties when the object is visually symmetric, but fail to do so when different forces are required at each digit. The ability to scale the overall gain of fingertip forces to object properties also implies that sensorimotor memories of overall force gain may be easily used, but memories related to how to distribute each digit force relative to the other may not. This is supported by studies where both the overall mass and the mass distribution of the object were varied [21], [22]. In these examples, following object translation to the contralateral hand or rotation in between successive lifts with the same hand, subjects scale their forces overall to be higher for heavier objects independent of mass distribution, but are unable to partition their forces to prevent tilt based on their awareness of the that distribution when it is asymmetric. This suggests that force distribution is largely reliant on sensorimotor memories from previous lifts. Thus on initial lifts with a novel object with asymmetric mass distribution or following object rotation/translation, absence of an immediate sensorimotor memory results in the inability to initially partition the forces. However, failure of using density cues for asymmetric but not symmetric mass distribution cannot be attributed solely to subjects’ difficulty with generating asymmetric forces: failure occurs following object rotation also when subjects could exert symmetrical fingertip load force by placing the digit non-collinearly on the object [23], [44]. Nevertheless, visual cues indicating asymmetrical CM location enabled subjects to modulate contact points on initial lifts, but object tilt still occurred, i.e., forces therefore were not correctly anticipated [45]. Further work is needed to understand the neural mechanisms underlying the failure to use perceived mass location cues for force control, and whether this failure would extend to contact location.
